# Impact of an mHealth App (Kencom) on Patients With Untreated Hypertension Initiating Antihypertensive Medications: Real-World Cohort Study

**DOI:** 10.2196/52266

**Published:** 2024-11-26

**Authors:** Koichiro Matsumura, Atsushi Nakagomi, Eijiro Yagi, Nobuhiro Yamada, Yohei Funauchi, Kazuyoshi Kakehi, Ayano Yoshida, Takayuki Kawamura, Masafumi Ueno, Gaku Nakazawa, Takahiro Tabuchi

**Affiliations:** 1 Department of Cardiology, Kindai University Faculty of Medicine, 377-2, Onhohigashi, Osakasayma, 5898511, Japan, 81 723660221; 2 Department of Social Preventive Medical Sciences, Center for Preventive Medical Sciences, Chiba University, Chiba, Japan; 3 Cancer Control Center, Osaka International Cancer Institute, Osaka, Japan; 4 Division of Epidemiology, School of Public Health, Tohoku University Graduate School of Medicine, Miyagi, Japan

**Keywords:** untreated hypertension, mobile health app, antihypertensive medication, cardiovascular disease, mHealth

## Abstract

**Background:**

To prevent the further development of cardiovascular diseases, it is a growing global priority to detect untreated hypertension in patients and ensure adequate blood pressure control via drug therapy. However, few effective tools that facilitate the initiation of antihypertensive medications among such patients have been identified.

**Objective:**

We aimed to determine whether a mobile health (mHealth) app facilitates the initiation of antihypertensive medications among patients with untreated hypertension.

**Methods:**

We analyzed a large longitudinal integrated database mainly comprised of data from middle-aged, employed people and their families. The database contained data from health checkups, health insurance claims, and the mHealth app kencom. kencom is used to manage daily life logs (eg, weight, number of steps) and to provide health information tailored to customers. Patients with untreated hypertension were identified using the baseline health checkup data, and follow-up health checkups were conducted to identify the rate of initiation of antihypertensive medications between mHealth app users and nonusers. Antihypertensive medication status was confirmed via a questionnaire administered during the medical checkup as well as a review of the health insurance claims database. We conducted a modified Poisson regression analysis, weighted by inverse probability of treatment weighting, to examine the effect of mHealth app usage on the initiation of antihypertensive medications. Additionally, data from four lifestyle questionnaires from the baseline and follow-up health checkups were collected to evaluate lifestyle modifications that could be attributed to the mHealth app.

**Results:**

Data were collected from 50,803 eligible patients (mean age 49, SD 9 years; men n=39,412, 77.6%; women n=11,391, 22.4%) with a median follow-up period of 3.0 (IQR 2.3‐3.1) years. The rate of initiation of antihypertensive medications was significantly higher in the mHealth app user group than in the nonuser group: 23.4% (3482/14,879) versus 18.5% (6646/35,924; *P*<.001), respectively. The risk ratio of mHealth app usage for initiated antihypertensive medications was 1.28 (95% CI 1.23‐1.33). Among those who did not intend to improve their lifestyle habits such as exercise and diet at baseline, the rate of lifestyle improvement at follow-up was compared between mHealth app users and nonusers, using data from the questionnaires; mHealth app users demonstrated a significantly higher rate of lifestyle changes than nonusers.

**Conclusions:**

For patients with untreated hypertension, the use of the mHealth app kencom, which was not dedicated to hypertension treatment, was associated with a higher initiation of antihypertensive medications.

## Introduction

Hypertension is a major risk factor for the development of cardiovascular and renal diseases, causing 8.5 million deaths annually worldwide [1,2]. According to a nationwide prospective survey of US adults (the Third National Health and Nutrition Examination Survey), patients with untreated hypertension in the United States have a 1.4-fold increased risk of all-cause mortality and a 1.77-fold increased risk of cardiac death compared to normotensive patients [3]. In a recent meta-analysis, a 5-mm Hg reduction in systolic blood pressure (SBP) reduced the risk of major cardiovascular events by approximately 10% [4]. Hypertension can be easily detected at community health care and primary care facilities and managed using inexpensive drug therapy [5]. Adequate therapeutic interventions that use antihypertensive medications can prevent the development of cardiovascular disease [4,6]. The number of patients with hypertension worldwide has doubled from 650 million in 1990 to 1.28 billion in 2019, with more than 700 million estimated untreated individuals [7]. To prevent the further development of cardiovascular diseases, it is a growing global priority to detect untreated hypertension in patients and ensure adequate blood pressure control via drug therapy [8]. Cooperative efforts, such as raising awareness of hypertension and improving access to affordable medical care, are needed to initiate therapeutic interventions for patients [9]. It is important that patients with documented hypertension understand that it is strongly associated with the development of future cardiovascular events and that adequate antihypertensive treatment can be a powerful preventive strategy. An insufficient understanding of antihypertensive treatment can reduce the number of health care visits, leaving hypertension untreated. In previous studies, patients with untreated hypertension who were younger and had a higher self-rated health status and no history of cardiovascular disease were more likely to not initiate hypertension treatment [10,11]. In addition, the absence of symptoms was a major reason why patients with untreated hypertension did not initiate treatment [11]. Helping such patients obtain adequate information about their own health and better understand medical information is important for their initiation of antihypertensive treatment [12].

Mobile health (mHealth) apps are software designed for smartphones and other mobile devices that focus on promoting health and wellness. Leveraging mobile device capabilities, such as sensors, connectivity, and user interfaces, provides a variety of health-related services and support. mHealth apps not only support disease prevention, management, and treatment but also support patients psychologically and in their decision-making [12]. Several recent reports recognized the blood pressure–reducing effects of digital therapeutic interventions [13,14]. In a randomized controlled trial, mHealth app users showed significantly improved blood pressure compared to nonusers [13]. Additionally, in a scoping review of patients with hypertension, a web-based diet and physical activity intervention program lowered blood pressure and improved communication between patients and health care providers, medication adherence rates, and the rate of medical visits [14]. These findings suggest that digital therapeutic interventions for patients with hypertension can lead to multifaceted lifestyle changes and nonpharmacologically improve blood pressure [12]. However, there is little evidence of the impact of mHealth apps on untreated hypertension. We hypothesized that an mHealth app may effectively change the behaviors of patients with untreated hypertension upon starting treatment. Accordingly, this study aimed to investigate whether an mHealth app would contribute to patients with untreated hypertension initiating drug therapy.

## Methods

### Databases

A retrospective cohort study design was used. We used large longitudinal databases provided by DeSC Healthcare Corporation (Tokyo, Japan). These databases consisted of three data sources: the Japanese health checkup database, the Japanese health insurance claims database, and the kencom database. The Japanese health checkup database consists of questionnaire results, physical examinations, biomarker measurements, and imaging examinations performed annually for the majority of adults living in Japan. The Japanese health insurance claims database records monthly information on patient demographics; diagnoses from the *International Classification of Diseases, Tenth Revision*; medical procedures; and medications. The kencom database is aggregated primarily based on daily physical activity data and app usage. The participants selected from these databases were those who resided in Japan and were members of the Social Insurance Labour Association, one of the main insurers of universal health insurance in Japan, and their family members. All participants downloaded and used the kencom app free of charge.

### Ethical Considerations

Informed consent was obtained for all the participants, including consent for secondary analysis. The anonymization of the data was based on an opt-out agreement between the user and the Social Insurance Labour Association, which notified the user of any data use and allowed them to request that the data be deleted. No compensation was paid to the participants who were surveyed. While each of the three databases was anonymized and stored separately, we combined the three into one database and conducted our analysis based on the unique numbers assigned to each participant. This study was approved by the ethics committees of Kindai University Hospital (R03-139).

### Outline of Kencom and Its Functionality

The mHealth app used in this study was kencom, developed by DeSC Healthcare Corporation and available on iOS and Android platforms. kencom manages daily life logs (eg, weight, number of steps) and provides health information tailored to its customers [15]. Steps are counted by the built-in pedometer on each smartphone, and data can be synchronized with kencom. Users can set daily physical activity goals and receive feedback based on achievements through self-checks. Users can also manually input their weight, blood pressure, and blood glucose levels into the app. Original health information on lifestyle and disease risk provided by kencom is peer reviewed by medical doctors and nutritionists.

### Target Population

**Figure 1.**
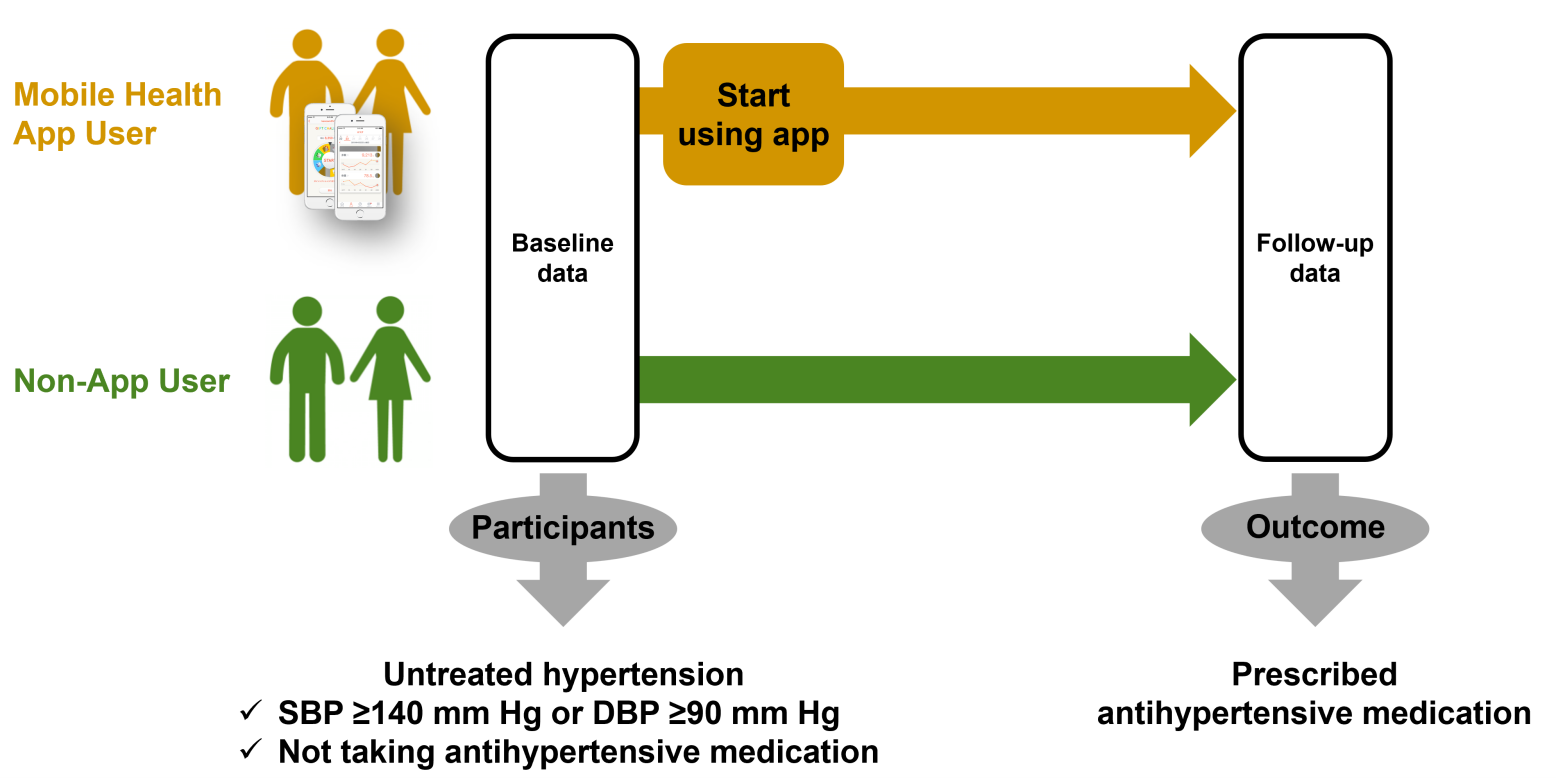
Study design flowchart. DBP: diastolic blood pressure; SBP: systolic blood pressure.

Out of 864,413 individuals whose data were collected between January 2016, when the kencom app service was launched, and September 2021, 8.3% (n=71,718) were identified as patients with untreated hypertension and were initially considered for the study. All participants who had downloaded the kencom app were automatically registered in the kencom database. The study required the participants who had downloaded kencom to have had their baseline health checkup data collected within 3 months prior to the time they downloaded the app. Data from follow-up health checkups conducted more than 1 year after the baseline health checkup were considered in the study. The following patients were excluded: those <20 years of age (79/71,718, 0.1%); those for whom blood laboratory data on triglycerides, high-density lipoprotein cholesterol, or fasting glucose were missing (418/71,718, 0.6%); those for whom no health checkup data within 3 months prior to downloading the app were available (5379/71,718, 7.5%); and those for whom no health checkup data >1 year after baseline were available (15,039/71,718, 21%). The final analysis categorized the remaining 50,803 patients into those who downloaded the kencom app (mHealth app users) and those who did not (nonusers) (Figure 1).

Patients with untreated hypertension were defined by the following criteria: those with SBP ≥140 mm Hg or diastolic blood pressure (DBP) ≥90 mm Hg who were not taking antihypertensive medications at the baseline medical checkup [16]. Antihypertensive medication status was confirmed via a questionnaire administered during the medical checkup as well as a review of the health insurance claims database. Antihypertensive drugs included thiazide diuretics, beta blockers, angiotensin-converting enzyme inhibitors, angiotensin receptor blockers, angiotensin receptor–neprilysin inhibitors, and calcium channel blockers.

### Primary Outcome

The primary outcome was the rate of initiation of antihypertensive medications at follow-up. Information regarding the initiation of antihypertensive medication use was collected from the health insurance claims database.

### Covariates

Age; sex; BMI; blood pressure; abdominal circumference; blood laboratory data; medical history; life history; and presence of metabolic syndrome, antihyperglycemic medications, and antihyperlipidemic medications were obtained from the baseline health checkup data. We used the Japanese criteria to identify metabolic syndrome [17], which included abdominal obesity (abdominal circumference ≥85 cm in men and ≥90 cm in women) as an essential criterion, as well as any two of the following three factors: dyslipidemia (triglycerides ≥150 mg/dL, high-density lipoprotein cholesterol level <40 mg/dL, or prescribed antihyperlipidemic medications), blood pressure ≥130/85 mm Hg or prescribed antihypertensive medications, and fasting glucose ≥110 mg/dL. Baseline blood pressure was graded according to the 2018 European Society of Cardiology/European Society of Hypertension guidelines: grade I, SBP 140‐159 mm Hg or DBP 90‐99 mm Hg; grade II, SBP 160‐179 mm Hg or DBP 100‐109 mm Hg; and grade III, SBP ≥180 mm Hg or DBP ≥110 mm Hg [18]. Following this, data were collected from four lifestyle questionnaires from the baseline and follow-up health checkups, addressing the following: light, sweaty exercise ≥1 year (Do you engage in light, sweaty exercise at least twice a week and for at least 1 year, for at least 30 minutes per session?); walking or an equivalent physical activity ≥1 hour/day (Do you walk or perform an equivalent physical activity in your daily life for at least 1 hour/day?); eating dinner within 2 hours before bedtime (Do you eat dinner within 2 hours before bedtime at least three times a week?); and intent to improve lifestyle habits such as exercise and diet (Are you intending to improve your lifestyle habits such as exercise and eating habits?). The responses to the final lifestyle questionnaire were as follows: no intention to improve (precontemplation), will improve within 6 months (contemplation), will improve within 1 month and have started gradually (preparation), have been working on improvement for 6 months or less (action), and have been working on improvement for more than 6 months (maintenance). The proportion of patients who responded “no” to each of the four lifestyle questionnaires at baseline but responded “yes” at follow-up was examined.

### Statistical Analysis

In the descriptive statistics employed in this study, means and SDs or median and IQRs were used for continuous variables, and numerical values and percentages were used for categorical variables. Intergroup equality was determined using the chi-square test for categorical variables. Inverse probability of treatment weighting (IPTW) was performed to create synthetic cohorts in which the treatment assignment was independent of the measured baseline covariates. IPTW attempts to address the selection of individuals for mHealth app usage by matching the characteristics that predict treatment status. It estimates the average treatment effect, surmising the effect of the treatment in a scenario in which everyone within the population is offered it. We conducted a logistic regression analysis in which the dependent variable was the mHealth app (binary variable) and the independent variables were all of the abovementioned covariates to calculate propensity scores. The propensity scores in this case were the conditional probability of an individual using kencom, Pr[A = 1|L], where A denotes treatment status and L represents all covariates. These propensity scores were computed for each participant, leading to the derivation of inverse probability of treatment weights of 1/Pr[A = 1|L] for mHealth app users and 1/(1 – Pr[A = 1|L]) for nonusers, which was calculated on 100 imputed datasets. After balancing the covariates between the control and treated groups (Table S1 in Multimedia Appendix 1), we calculated the risk differences, which estimated the average of the predicted changes in the probability of initiating antihypertensive medications, and the risk ratios using a modified Poisson regression analysis weighted by IPTW. Four sensitivity analyses were performed to examine the heterogeneity of associations. First, a subgroup analysis was conducted to evaluate the risk ratios for all covariates. Second, the rate of initiation of antihypertensive medications between mHealth app users and nonusers was assessed and stratified by hypertension classification. Third, as mHealth app users were more likely than nonusers to be aware of the need to improve their lifestyle at baseline, responses collected at baseline to the questionnaire on intent to improve lifestyle habits such as exercise and diet were accordingly stratified into three groups: those that reported (1) action or maintenance, (2) preparation, and (3) precontemplation or contemplation. Finally, since kencom regularly promoted exercise campaigns (Arukatsu events), the mHealth user group was stratified by whether or not they participated in the campaigns.

In this study, there were variables with missing values. To address the potential bias resulting from missing data, we conducted multiple imputations using the Markov Chain Monte Carlo method under the assumption that data were missing at random conditions associated with all variables [19]. After generating 100 imputed datasets using all variables, we performed the analyses described above and combined the effect estimates. All statistical analyses were performed using Stata 17.0 software (StataCorp LLC). A 2-tailed α value of .05 was considered statistically significant.

## Results

### Patient Characteristics

In total, 50,803 patients (mHealth app users n=14,879, 29.3%; nonusers n=35,924, 70.7%) were included in the final cohort. The median observation period was 3.0 (IQR 2.3‐3.1) years. Patient backgrounds are shown in Table 1.

The mean patient age was 49 (SD 9) years, and 77.6% (39,412/50,803) of the patients were male and 22.4% (11,391/50,803) were female. Metabolic syndrome was present in one-fourth of all patients. Responses to the questionnaire on intent to improve lifestyle habits such as exercise and diet are shown under “Stage of health behavior changes.” The proportion of patients who answered “action” and “maintenance,” that is, who were already implementing healthy behavior changes to improve their lifestyle, was approximately one-fourth of the overall patients and was higher among mHealth app users (3589/12,792, 28.1%) compared to nonusers (7133/29,133, 24.5%).

**Table 1.** Baseline characteristics.

Patient characteristics	Entire cohort, n	mHealth^a^ app users	Nonusers
Age (years), mean (SD)	50,803	48 (8)	50 (10)
Sex	50,803		
Male, n/N (%)		12,982/14,879 (87.3)	26,430/35,924 (73.6)
Female, n/N (%)		1897/14,879 (12.7)	9494/35,924 (26.4)
BMI ≥25.0 kg/m^2^, n/N (%)	50,801	6398/14,879 (43.0)	14,353/35,922 (40.0)
Waist circumference (cm), mean (SD)	49,751	86 (10)	86 (10)
SBP^b^ (mm Hg), mean (SD)	50,803	143 (11)	144 (11)
DBP^c^ (mm Hg), mean (SD)	50,803	93 (8)	91 (9)
Hypertension classification, n/N (%)	50,803		
Grade I		12,459/14,879 (83.7)	30,221/35,924 (84.1)
Grade II		2045/14,879 (13.7)	4710/35,924 (13.1)
Grade III		375/14,879 (2.5)	993/35,924 (2.8)
Laboratory data, mean (SD)			
Triglyceride (mg/dL)	50,803	138 (111)	135 (112)
HDL^d^ cholesterol (mg/dL)	50,802	60 (16)	61 (17)
Fasting blood glucose (mg/dL)	43,425	99 (18)	100 (19)
Metabolic syndrome, n/N (%)	43,425	3441/13,550 (25.4)	7193/29,875 (24.1)
Receiving antihyperglycemic medications, n/N (%)	50,802	375/14,879 (2.5)	1119/35,923 (3.1)
Receiving antihyperlipidemic medications, n/N (%)	50,801	920/14,879 (6.2)	2572/35,922 (7.2)
History of cardiovascular disease, n/N (%)	42,511	188/13,020 (1.4)	490/29,491 (1.7)
History of stroke, n/N (%)	42,508	77/13,018 (0.6)	200/29,490 (0.7)
Current smoking, n/N (%)	50,793	3638/14,876 (24.5)	10,039/35,917 (28.0)
Alcohol consumption, n/N (%)	44,157	4863/13,414 (36.3)	9439/30,743 (30.7)
Stage of health behavior changes, n/N (%)	41,925		
Precontemplation		2461/12,792 (19.2)	7013/29,133 (24.1)
Contemplation		4787/12,792 (37.4)	10,523/29,133 (36.1)
Preparation		1955/12,792 (15.3)	4464/29,133 (15.3)
Action		1491/12,792 (11.7)	2890/29,133 (9.9)
Maintenance		2098/12,792 (16.4)	4243/29,133 (14.6)

a mHealth: mobile health.

b SBP: systolic blood pressure.

c DBP: diastolic blood pressure.

d HDL: high-density lipoprotein.

### Primary End Point

Data from the follow-up health checkups and the health insurance claims database were used to assess the rate of antihypertensive medication use. The rate of mHealth app users who initiated antihypertensive medications was 23.4% (3482/14,879), while the rate of nonusers who initiated the antihypertensive medications was 18.5% (6646/35,924) (*P*<.001). As seen, mHealth users had a significantly higher rate of initiation than nonusers.

### Average Treatment Effect of Kencom Usage on the Initiation of Antihypertensive Medications

We then estimated the average treatment effects of mHealth app usage on the initiation of antihypertensive medication use. In the IPTW cohort, the estimated risk of initiating antihypertensive medications was 0.234 (95% CI 0.227-0.241) and 0.183 (95% CI 0.179-0.187) in the treated and control groups, respectively. The risk ratio was 1.28 (95% CI 1.23-1.33), and the risk difference was 0.051 (95% CI 0.043-0.059).

### Lifestyle Modifications

Among those who did not intend to improve their lifestyle habits at baseline, the rate of lifestyle improvement at follow-up was compared between mHealth app users and nonusers (Table 2). The rate of patients who performed light, sweaty exercise for ≥1 year at follow-up was 12.4% (1594/12,906) among mHealth app users versus 10% (2889/28,878) among nonusers (*P<*.001). Likewise, the rate of walking or performing an equivalent physical activity ≥1 hour/day was 15.7% (2042/12,993) among mHealth app users versus 12.8% (3754/29,319) among nonusers (*P<*.001). The percentage of respondents who did not eat dinner within 2 hours before bedtime was 13.7% (1850/13,533) among mHealth app users versus 10.3% (3256/31,591) among nonusers (*P<*.001). An increase in the intent to improve lifestyle habits such as exercise and diet was noted among 18.9% (2397/12,685) of mHealth app users versus 14.4% (4121/28,559) of nonusers (*P*<.001). All questionnaires indicated that mHealth app users demonstrated a significantly higher rate of lifestyle changes at follow-up than nonusers.

**Table 2.** Rate of patients with improved lifestyle changes.

Lifestyle habits	mHealth^a^ app users, n/N (%)	Nonusers, n/N (%)	*P* value
Performed light, sweaty exercise ≥1 year	1594/12,906 (12.4)	2889/28,878 (10)	<.001
Walked or an equivalent physical activity ≥1 hour/day	2042/12,993 (15.7)	3754/29,319 (12.8)	<.001
Did not eat dinner within 2 hours before bedtime	1850/13,533 (13.7)	3256/31,591 (10.3)	<.001
Intent to improve lifestyle habits such as exercise and diet	2397/12,685 (18.9)	4121/28,559 (14.4)	<.001

a mHealth: mobile health.

### Sensitivity Analyses

**Figure 2.**
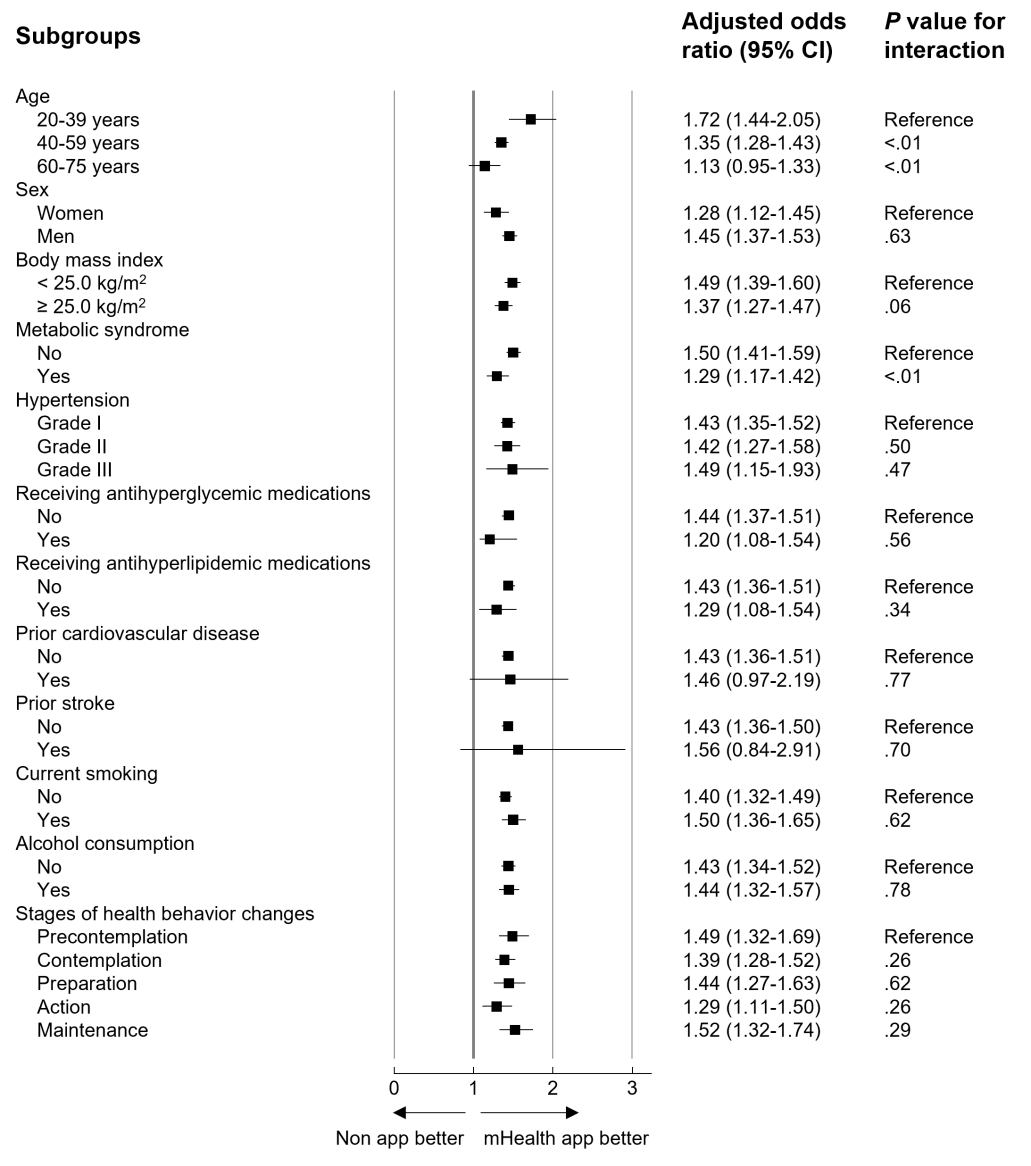
Primary outcome analyzed according to subgroup in the inverse probability of treatment weighting cohort. mHealth: mobile health.

In the subgroup analyses, there were significant interactions between mHealth app usage and the initiation of antihypertensive medications for the subgroups of age, metabolic syndrome, and hypertension classification (Figure 2). The beneficial effects of homogeneity were shown by sex, BMI, smoking, alcohol consumption, and the stage of health behavioral changes.

When stratified by hypertension classification, mHealth app users were significantly more likely to initiate antihypertensive medication use regardless of severity (mHealth app users vs nonusers: grade I, 1885/10,890, 17.3% vs 3340/25,518, 13.1%; grade II, 1337/3610, 37% vs 2734/9403, 29.1%; grade III, 252/379, 66.5% vs 571/1003, 56.9%; Figure S1 in Multimedia Appendix 1).

In the mHealth app user group, we evaluated the rate of initiation of antihypertensive medications among those who participated in the exercise campaigns (Arukatsu events) and those who did not. The results showed that 23% (1340/5820) of those who participated in the exercise campaigns and 23.6% (2134/9059) of nonparticipants started antihypertensive medications, with comparable rates in both groups (*P*=.45).

In response to the questionnaire on intent to improve lifestyle habits such as exercise and diet asked at baseline, the rate of initiated antihypertensive medications was compared between mHealth app users and nonusers, then stratified into three groups. Among those who responded “precontemplation” or “contemplation,” the rate of initiated antihypertensive medications was significantly higher among mHealth app users (865/3589, 24.1%) compared with nonusers (1342/7133, 18.8%) (*P<*.001). Similarly, in those who responded with “preparation,” the rate of initiated antihypertensive medications was 25.6% (500/1955) among mHealth app users versus 19.4% (864/4464) among nonusers (*P*<.001). In those who responded with “action” or “maintenance,” 23.5% (1700/7248) of mHealth app users versus 18% (3161/17,536) of nonusers (*P<*.001) initiated hypertensive medications. As seen, the rate of initiation was significantly higher among mHealth app users in all three groups.

## Discussion

### Overview

In this study, we examined whether an mHealth app (kencom) that is not dedicated to hypertension treatment could help promote the initiation of antihypertensive medications among patients with untreated hypertension, mainly middle-aged, employed people and their families. mHealth app users had higher rates of initiated antihypertensive medication use than nonusers, with the use of kencom being associated with the initiation of antihypertensive medications in the multivariable analysis. In the group of patients with low motivation to improve their lifestyle at baseline, a significantly greater number of mHealth app users had improved their lifestyle at follow-up than nonusers. The subgroup analysis suggests that kencom may have had a greater impact on the initiation of antihypertensive medications in younger patients or in those without metabolic syndrome. Regardless of baseline blood pressure levels or the motivation to make lifestyle changes though, mHealth app users consistently initiated antihypertensive medications at higher rates. There are two steps to obtaining antihypertensive medications: the first step is to visit a medical care institution, and the second is to be examined by a physician and begin drug treatment. Therefore, the kencom app did not directly influence the initiation of antihypertensive medications. However, we believe that the results of this study suggest that an mHealth app may have some influence on the changes in health-related behaviors of patients with untreated hypertension, and may help increase their rate of visiting a medical institution.

Health literacy refers to the ability to obtain, read, understand, and use medical information to make informed health-related decisions, and it plays an important role in the implementation of medication therapy [20]. People with higher health literacy are more likely to understand the risks associated with hypertension and the importance of regular blood pressure monitoring, healthy lifestyle habits, and medical care [20]. They are also more likely to understand the benefits and potential side effects of different treatments and make informed decisions regarding their health care. In contrast, individuals with low health literacy may not fully understand the risks associated with hypertension, how to measure blood pressure, or how to manage their condition by developing healthy lifestyle habits. Consequently, hypertension may remain untreated, leading to serious health problems [10]. Therefore, health care providers need to focus on promoting pharmacotherapeutic interventions for hypertension and improving health literacy to help patients achieve better blood pressure control and ensure the long-term prevention of cerebrovascular diseases.

mHealth apps have been reported to effectively improve health literacy and adherence [21-23]. In our study, mHealth app users improved their exercise and eating habits at a higher rate than nonusers, which may have resulted from the app’s ability to improve health literacy. This change in patient perception may have facilitated hypertension treatment. There are various interventions to improve adherence to the treatment of chronic diseases, and mHealth apps promote several, including informational, behavioral, and social interventions [10]. They provide interactive education and obtain patient backgrounds, which allow personalized intervention through guidance and reviews. Additionally, mHealth apps support self-planning and share the user’s goal of improving their blood pressure and lifestyle. For interventions to be effective, it is important to have continuous and sustained contact with patients and combine strategies tailored to their needs; methods that include multiple interventions have a greater effect on adherence. Therefore, mHealth apps can be considered effective intervention tools.

Previous studies have reported that patients who are less likely to initiate antihypertensive treatment are those who are younger; are in better health; do not have obesity, a history of cardiovascular disease, or diabetes; or do not visit primary care physicians [11]. These patients are a common subgroup with an inadequate awareness of hypertension and poor blood pressure control [16]. In our study, mHealth app users had a higher rate of initiating antihypertensive medications compared to nonusers in these subgroup populations. The mHealth app was also found to be more effective in initiating antihypertensive medications in younger patients, possibly because they were less resistant to and more familiar with using mHealth apps than older patients [24]. Furthermore, the older population has a higher prevalence of chronic diseases than the younger population and may be more likely to visit a hospital and initiate treatment regardless of their mHealth app usage. These findings suggest that mHealth apps are more effective in patients with no history of chronic diseases, who are at a greater distance from their health care providers.

### Strengths and Limitations

Most existing research on mHealth apps for hypertensive patients is dominated by studies reporting the effect of mHealth app use on blood pressure reduction and medication adherence in patients who have visited a health care provider for hypertension [25]. However, patients with hypertension identified in the preliminary stages of visiting a health care provider do not tend to visit a hospital for further care thereafter, remaining untreated [26,27]. Therefore, it is important that hypertension is detected during health checkups by local health care providers before the patient is referred to a medical facility for treatment. Our study is of high clinical relevance, as it highlights the impact of mHealth apps on untreated hypertensive patients in the preliminary stage of visiting a medical facility. Furthermore, the analyzed population mainly consisted of middle-aged, employed people and their families, who would fully benefit from the early initiation of antihypertensive treatment. Our cohort study was conducted using a fairly large sample size, as compared to others that have investigated the relationship between mHealth app usage and hypertension treatment. The patients’ antihypertensive medication statuses were collected from receipt data and were highly accurate. These results are of social significance, as we have demonstrated that an mHealth app such as kencom helps patients with untreated hypertension initiate antihypertensive medications.

This study has several limitations. First, the participants were primarily healthy Japanese individuals; thus, our results may not be generalizable to other populations. Second, we could not completely exclude the possibility of unmeasured confounding factors. However, in the sensitivity analyses, mHealth apps showed good homogeneity and randomized controlled trials were required to infer the causal effects of mHealth app use on health outcomes. Third, the timing between when the kencom app was downloaded and when the baseline health checkup was conducted differed (ie, a gap of up to 3 months). Thus, patients with untreated hypertension classified as mHealth app users may have included those who visited health care providers on their own and initiated antihypertensive medications before downloading the app. Therefore, the effect of the mHealth app on initiating antihypertensive medications through prospective trials warrants further investigation. Fourth, kencom encourages behavior changes through an incentive system. This study did not examine the relationship between this system and the initiation of antihypertensive medications. Fifth, the extent to which mHealth app users utilized the app after downloading it was unknown. Therefore, we could not examine the association between the frequency of app use and initiation of antihypertensive medications. Finally, this study analyzed the impact of the kencom app on patients with untreated hypertension; however, it is unclear whether using other mHealth apps would lead to similar findings.

### Conclusions

This study suggests that among patients with untreated hypertension, mainly composed of middle-aged, employed people and their families, the use of mHealth apps in managing daily life logs (eg, weight, number of steps) and providing health information tailored to customers may be an effective means of facilitating the initiation of antihypertensive medications. Younger patients or those without metabolic syndrome may benefit more from mHealth app usage. Promoting antihypertensive treatment in the working population could reduce the development of cardiovascular diseases.

## Supplementary material

10.2196/52266 Multimedia Appendix 1 Supplementary material.

## Abbreviations

DBP: diastolic blood pressure
IPTW: inverse probability of treatment weighting
mHealth: mobile health
SBP: systolic blood pressure
