# Supplementary material for: Impact of an mHealth App (Kencom) on Patients With Untreated Hypertension Initiating Antihypertensive Medications: Real-World Cohort Study
Source: JMIR Cardio. 2024 Nov 26;8:e52266. doi: 10.2196/52266 (PMC11612529; doi:10.2196/52266)
Supplement: Multimedia Appendix 1 [file cardio-v8-e52266-s001.doc]

**Supplementary Table S1.** Standardized mean difference of variables between original and IPTW cohorts.

|  | **Original** | **Weighted** |
| --- | --- | --- |
| Age (years) | -0.194 | -0.059 |
| Men | 0.35 | 0.015 |
| BMI ≥25.0 kg/m^2^ | 0.062 | 0.013 |
| Metabolic syndrome | 0.022 | 0.007 |
| Hypertension |  |  |
| Grade I | -0.011 | -0.001 |
| Grade II | 0.019 | 0 |
| Grade III | -0.015 | 0.002 |
| Receiving antihyperglycemic medications | -0.036 | -0.005 |
| Receiving antihyperlipidemic medications | -0.039 | -0.015 |
| History of cardiovascular disease | -0.009 | -0.004 |
| History of stroke | -0.005 | -0.002 |
| Current smoking | -0.08 | 0.012 |
| Alcohol consumption | 0.109 | -0.002 |
| Stage of health behavior changes |  |  |
| Precontemplation | -0.109 | 0.002 |
| Contemplation | 0.033 | 0.005 |
| Preparation | -0.013 | 0.003 |
| Action | 0.039 | 0 |
| Maintenance | 0.061 | -0.012 |

BMI: body mass index; IPTW: inverse probability of treatment weighting.


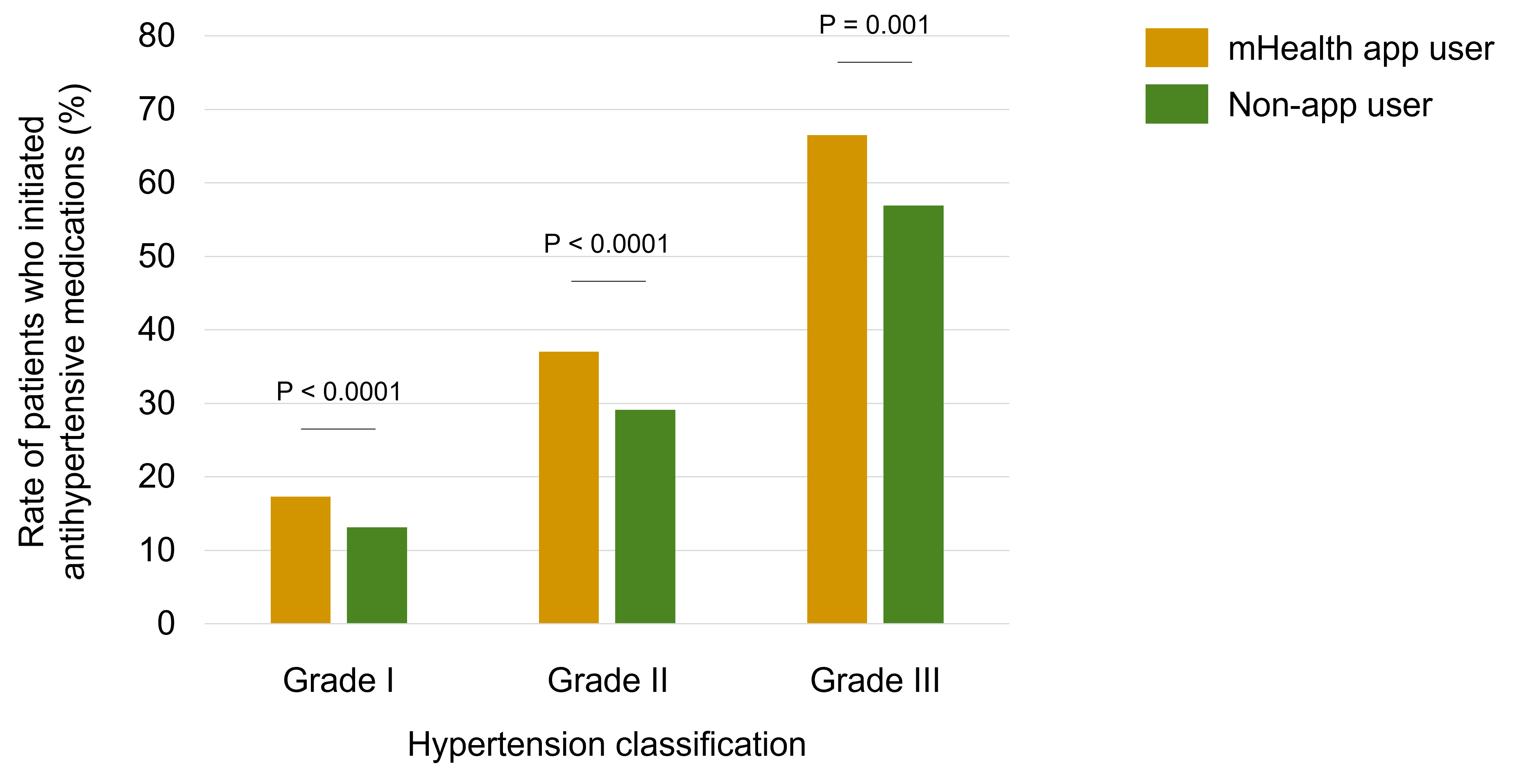


**Supplementary Figure S1.** Patients who initiated antihypertensive medications according to hypertension classification. Grade I: SBP 140-159 mm Hg or DBP 90-99 mm Hg, Grade II: SBP 160-179 mm Hg or DBP 100-109 mm Hg, and Grade III: SBP ≥180 mm Hg or DBP ≥110 mm Hg.

DBP: diastolic blood pressure; SBP: systolic blood pressure.
